# Supplementary material for: DNA double-strand break movement in heterochromatin depends on the histone acetyltransferase dGcn5
Source: Nucleic Acids Res. 2024 Sep 11;52(19):11753–67. doi: 10.1093/nar/gkae775 (PMC11514474; doi:10.1093/nar/gkae775)
Supplement: gkae775_Supplemental_Files [file gkae775_supplemental_files.zip › Kendek_Sandron_Supplementary_figures.pdf]

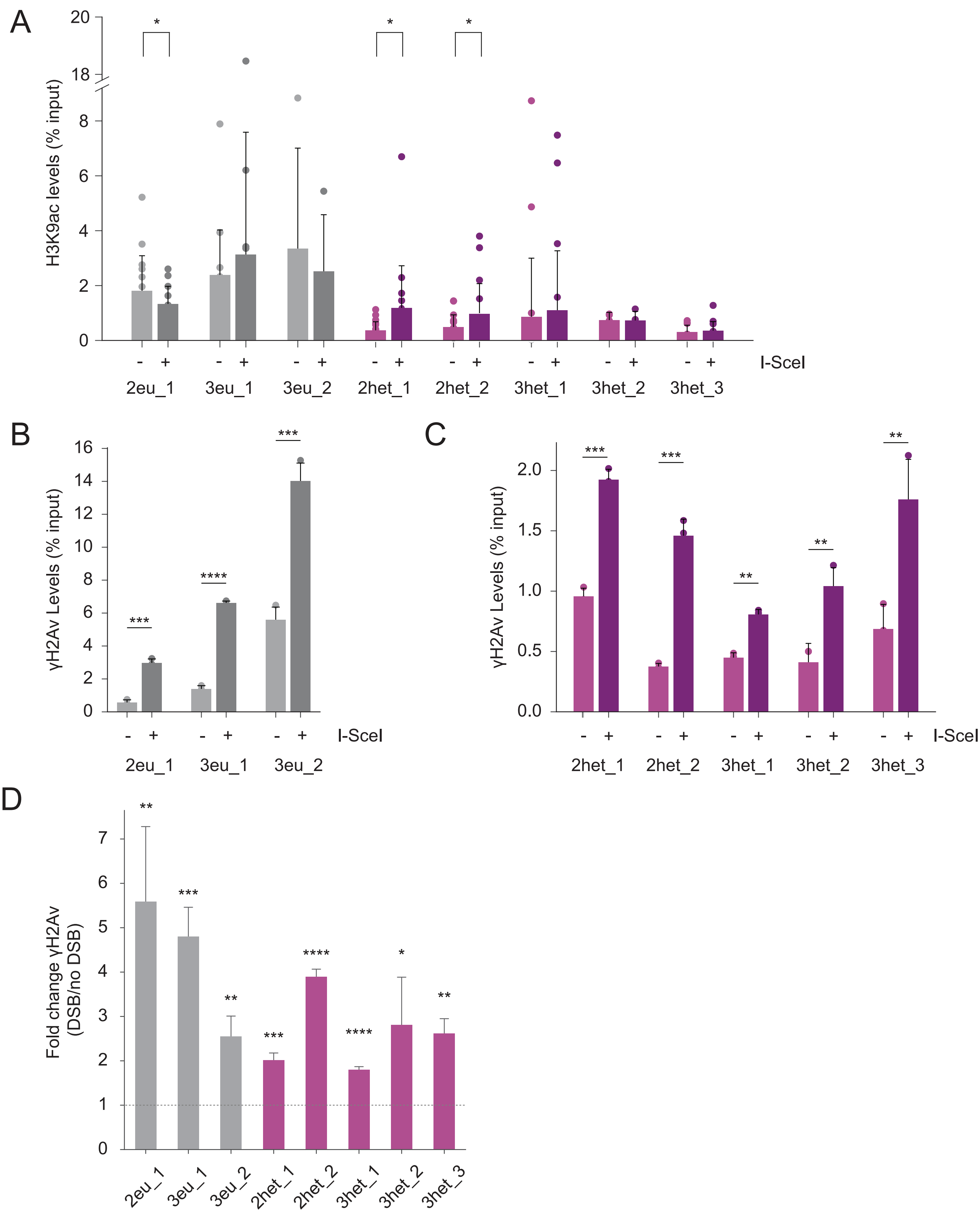

Figure S1. H3K9ac levels increase at heterochromatic DSBs. A. ChIP-qPCR result for H3K9ac in three euchromatic and five heterochromatic DR-*white* lines, with (+DSB, +I-SceI) and without (-DSB, -I-SceI) DSB induction. Graph indicates the percentage of input (normalized to internal *yellow* control gene) of H3K9ac from the same experiments as in *Fig. 1C*. Error bars represent mean +SD from ≥4 independent experiments. **B, C.** ChIP-qPCR result for γH2Av in three euchromatic (*B*) and five heterochromatic (*C*) DR-*white* lines, with (hsp.I-SceI) and without (no hsp.I-SceI) DSB induction. Graph indicates the percentage of input (normalized to internal *yellow* control gene) from the same experiments as in *Fig. 1C*. **D.** Fold change γH2Av is calculated by dividing the γH2Av enrichment levels in the damaged (+ DSB, + I-SceI) samples by those in the controls (-DSB, no I-SceI). The grey dotted line represents a fold change of 1, in which γH2Av levels are unchanged upon DSB induction. For *B*, *C*, and *D*, error bars represent mean +SD from 3 independent experiments. (*) P-value≤0.05, (**) P-value≤0.01, (***) P-value≤0.001, (****) P-value≤0.0001, t-test.

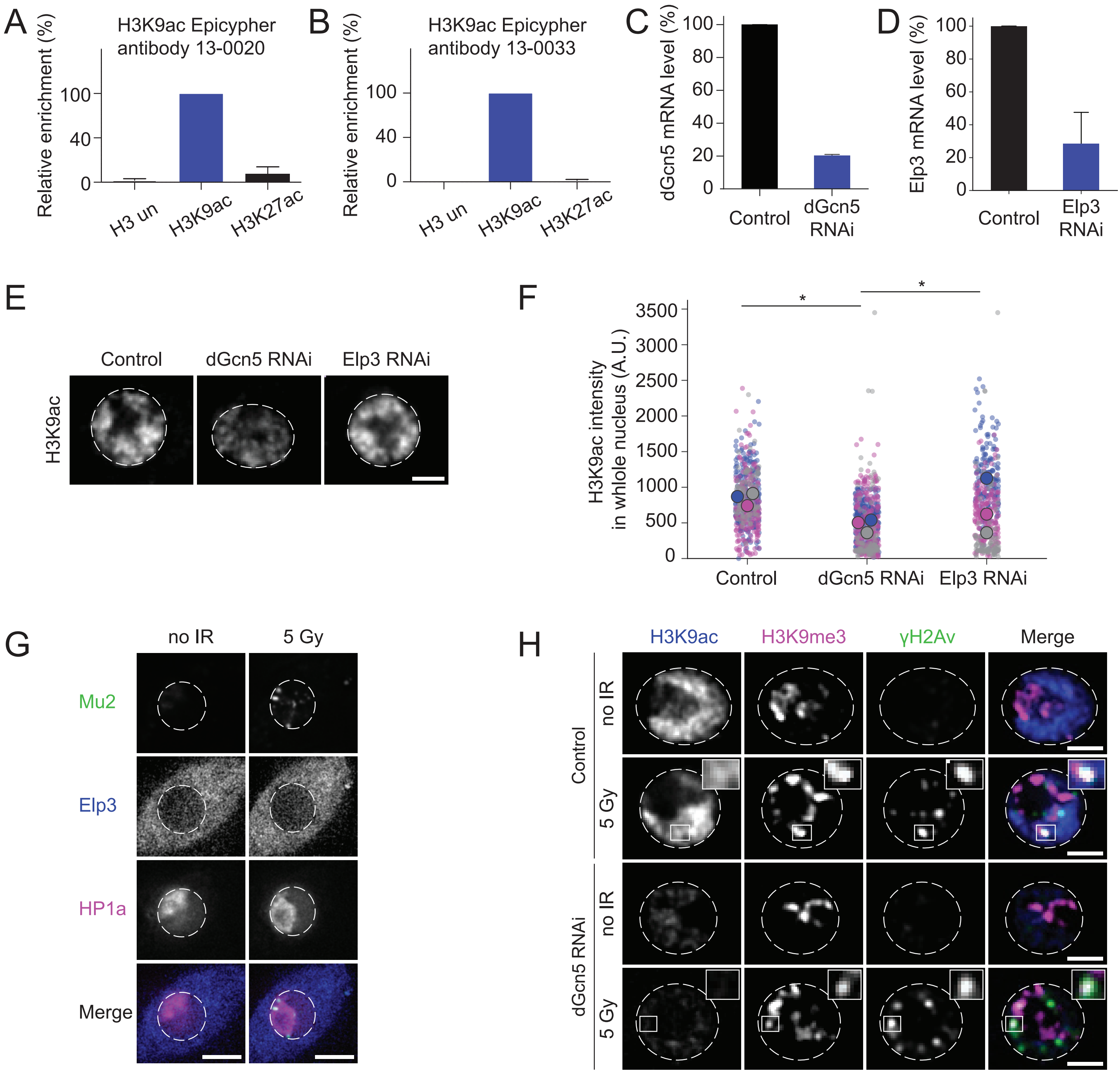

Figure S2. dGcn5 controls H3K9ac levels in *Drosophila*. A, B. Specificity test for two anti-H3K9ac antibodies from Epicypher (*A*, lot number 13-0020, *B*, lot number 13-0033). Specificity was determined using the SNAP-ChIP K-AcylStat nucleosome panel from Epicypher. Error bars indicate mean +SD from ≥3 independent experiments. H3 un = unmodified H3. **C, D.** Normalized expression levels of dGcn5 (*C*) and Elp3 (*D*) in control (*yellow* dsRNA) and HAT-depleted Kc cells, determined by Reverse Transcription followed by quantitative PCR (RT-qPCR). Error bars represent mean +SD from 3 independent experiments. **E.** Immunofluorescence staining for H3K9ac in control, dGcn5-depleted and Elp3-depleted S2 cells. Dashed lines enclose the nuclei. **F.** Quantification of absolute nuclear H3K9ac intensity levels in control, dGcn5-depleted and Elp3-depleted S2 cells. Each bigger circle (grey, blue, magenta) represents the average intensity within one experiment. Smaller circles represent individual cells within one experiment. **G.** Representative time-lapse images of non-irradiated and 5 Gy-irradiated Kc cells with fluorescently tagged Mu2 (green, DSB marker), Elp3 (blue) and HP1a (magenta, heterochromatin marker). Images were taken 10 minutes after IR. Dashed lines enclose the nuclei. Scale bars = 5 μm. **H.** Representative immunofluorescence images for the quantification shown in *Fig. 1E, F*. Non-irradiated and irradiated control (*yellow* dsRNA) and dGcn5-depleted Kc cells were stained for H3K9ac (blue), H3K9me3 (magenta, heterochromatin marker) and γH2Av (green, DSB marker). Dashed lines enclose the nuclei. Insets are zoom-in views of H3K9ac and H3K9me3 levels at γH2AV foci. Irradiated cells were fixed 5-10 minutes after damage induction. For *E* and *H*, scale bar = 2 μm. For *F*, (*) P-value≤0.05, one way ANOVA followed by Tukey's multiple comparison. If not shown, P-value not significant (>0.05).

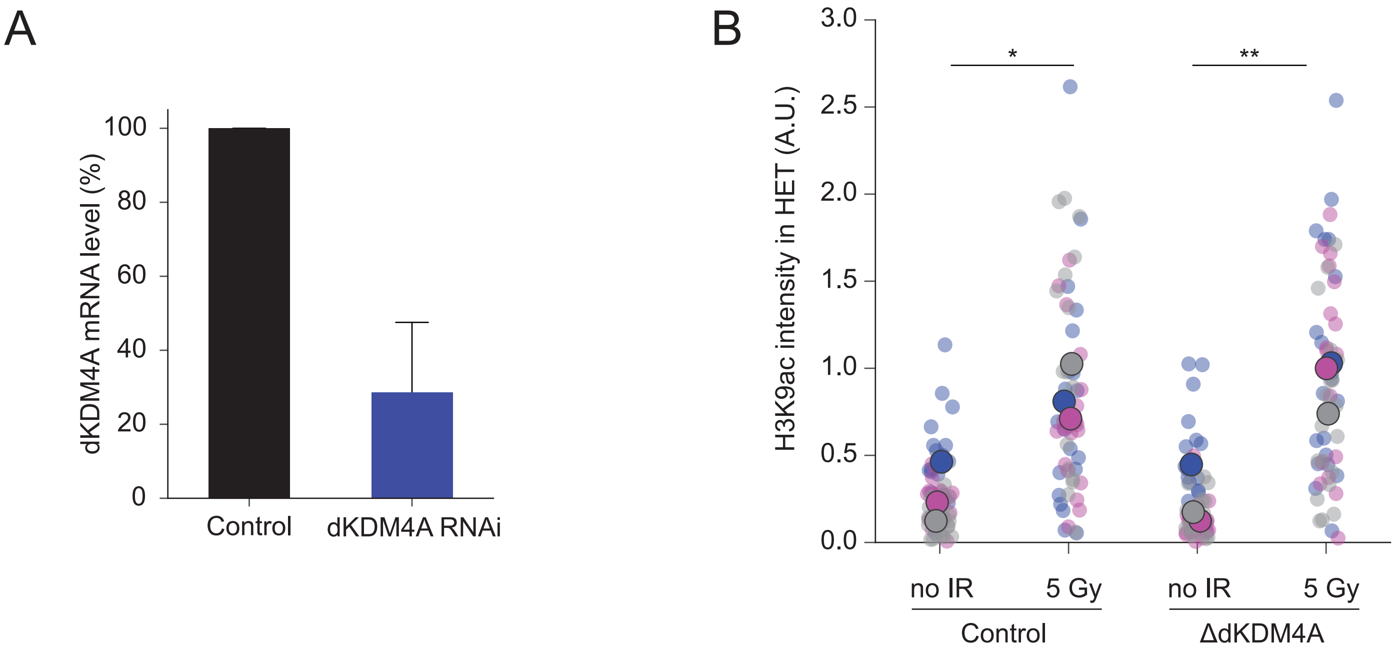

Figure S3. Deposition of new H3K9ac marks at heterochromatic DSBs is independent of dKDM4A. A. Normalized expression levels of dKDM4A in control (*yellow* dsRNA) and dKDM4A-depleted Kc cells, determined by RT-qPCR. Error bars represent mean +SD from 3 independent experiments. **B.** Quantification of H3K9ac intensity levels at DSBs in heterochromatin (DAPI bright) in non-irradiated versus irradiated control and ΔdKDM4A larval wing disc cells. For non-irradiated cells, H3K9ac signal was quantified in the entire DAPI bright (heterochromatin) area. Irradiated cells were fixed 5-10 minutes after damage induction. The graph represents 3 independent experiments per condition. Each big circle (grey, blue, magenta) represents the average intensity within one experiment. Small circles represent individual cells within one experiment. (*) P-value≤0.05, (**) P-value ≤ 0.01, one way ANOVA followed by Tukey's multiple comparison.

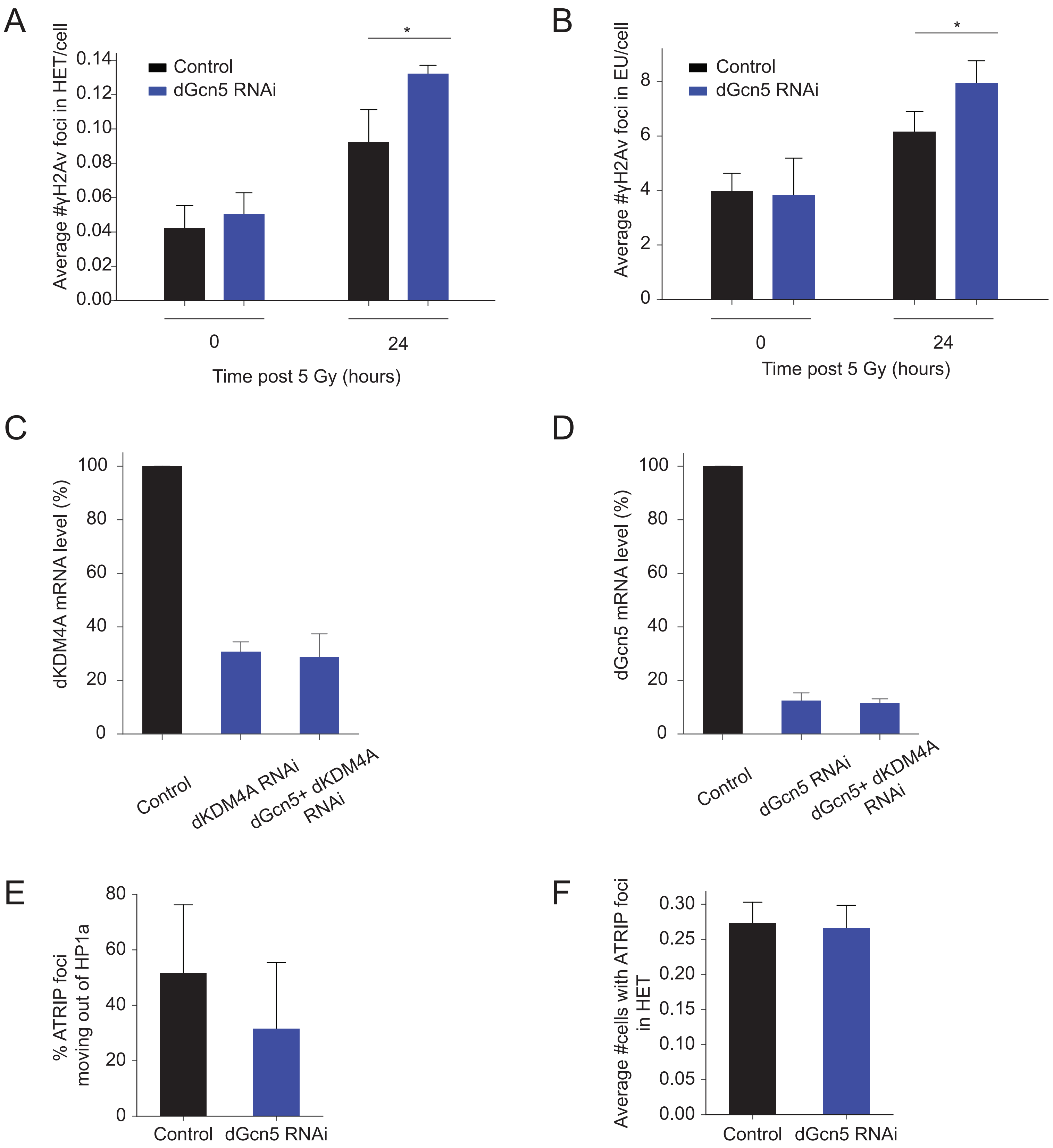

Figure S4. dGcn5 promotes ATRIP foci movement outside heterochromatin. A, B. Quantification of γH2Av foci inside DAPI bright (*A*) and DAPI weak (*B*) in non-irradiated versus 24 hours after irradiation control (*yellow* dsRNA) and dGcn5-depleted Kc cells. HET = heterochromatin, EU = euchromatin. Error bars represent mean +SD from 3 independent experiments. **C, D.** Normalized expression levels of dKDM4A (*C*) and dGcn5 (*D*) in control (*yellow* dsRNA), dKDM4A- or dGcn5-depleted and dKDM4A+dGcn5 depleted Kc cells, determined by RT-qPCR. Error bars represent mean +SD from 3 independent experiments. **E.** Quantification of the percentage of ATRIP foci that eventually move out of HP1a domain as depicted in *Fig. 3B*. **F.** Quantification of the average number of cells with ATRIP foci inside HP1a domain upon control (*yellow* dsRNA) or dGcn5 depletion within the window of time imaged (120 minutes). Error bars represent mean +SD from 3 independent experiments. For *A*, *B*, *E* and *F*, (*) P-value≤0.05, paired t-test. If not shown, P-value not significant (>0.05).

A

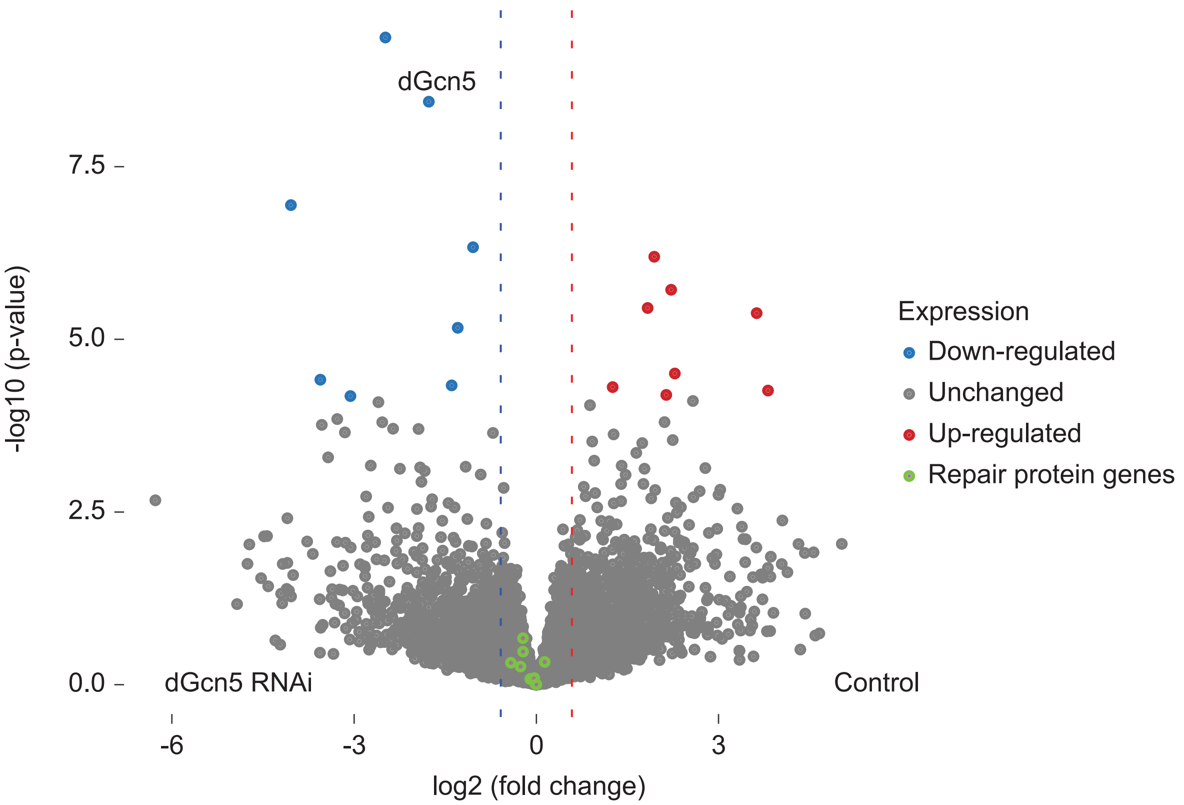

B

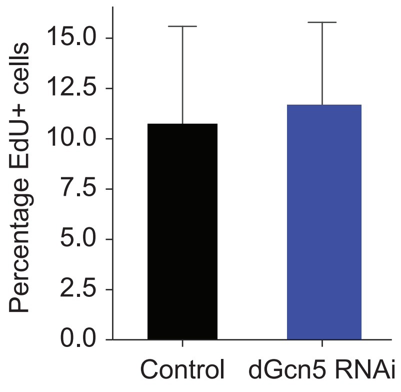

Figure S5. dGcn5 knockdown does not affect cell cycle stage or transcription of DSB repair genes. A. Volcano plot for bulk RNA-sequencing of Kc cells depleted of dGcn5 or *yellow* (control). See *Table S2-4* for more details. **B.** Quantification of the percentage of EdU positive (S-phase) Kc cells upon dGcn5 and *yellow* (control) knockdown. Error bars represent mean +SD from 3 independent experiments.

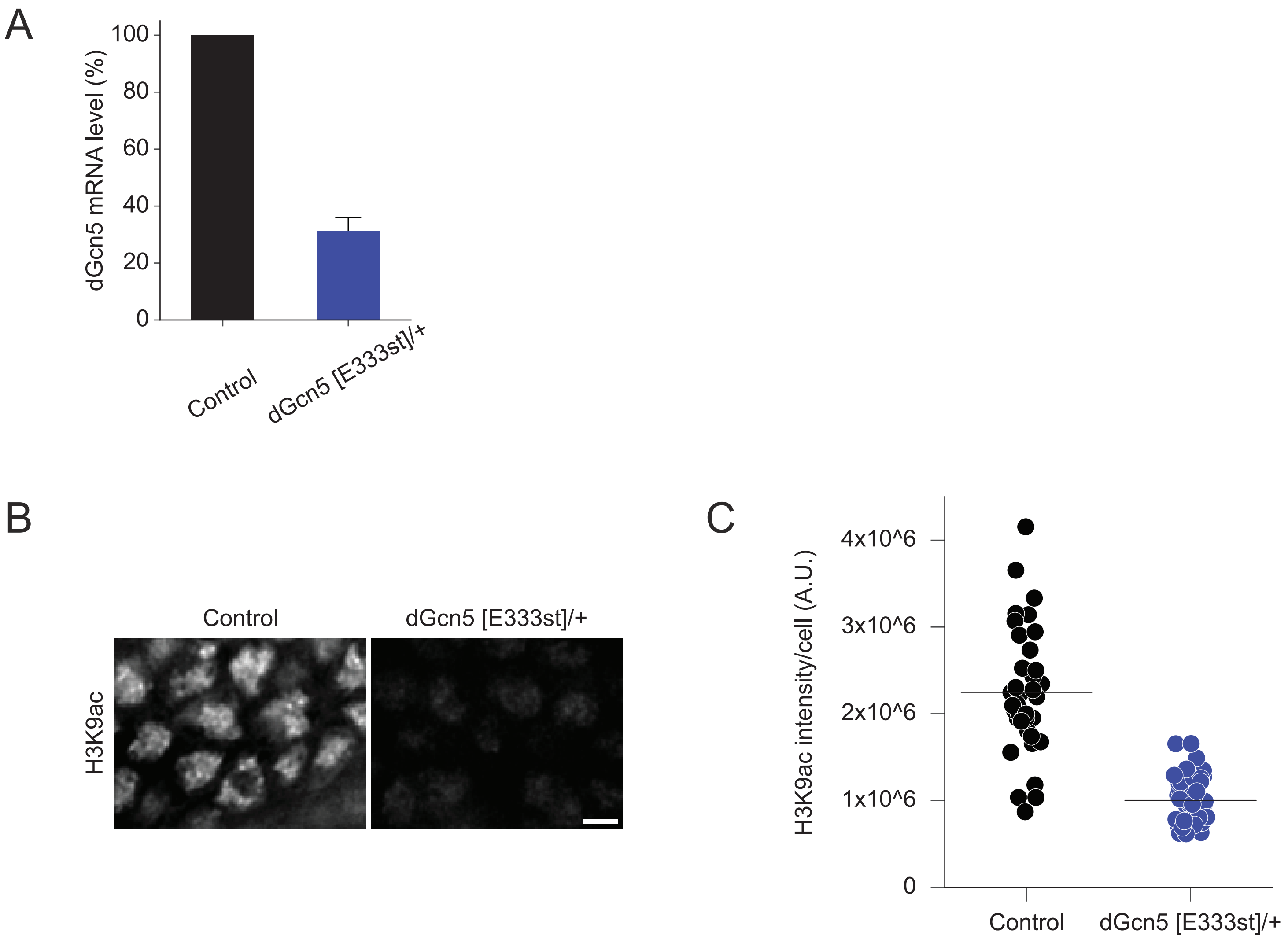

Figure S6. dGcn5 [E333]/+ mutant larval tissue has reduced H3K9ac nuclear levels. A. Normalized expression levels of dGcn5 in control (OregonR) and dGcn5 [E333st]/+ third instar larval tissues as determined by RT-qPCR. Error bar indicates mean +SD from 3 independent experiments. **B.** Representative images of *Drosophila* wing discs dissected from third instar larvae of OregonR (Control) or dGcn5 [E333st]/+ and immuno-stained for H3K9ac. Scale bar = 2 μm. **C.** Quantification of H3K9ac intensity per cell for the wing discs depicted in *B*. Lines represent mean intensity.
